# Editorial: Epigenetics and metabolism: a closed liaison in tumor cells

**DOI:** 10.3389/freae.2026.1886632

**Published:** 2026-06-03

**Authors:** Serena Castelli, Nicolò Mangraviti, Rohan Kodgule

**Affiliations:** 1 Department of Human Science and Promotion of Quality of Life, San Raffaele Telematic University, Rome, Italy; 2 New York University Grossman School of Medicine, New York, NY, United States; 3 Goldfarb School of Nursing at Barnes-Jewish College, St. Louis, MO, United States

**Keywords:** chromatin regulation, epigenetics, metabolism, ncRNAs (non coding RNAs), tumor

The interplay between metabolism and epigenetics has emerged as one of the most dynamic and rapidly evolving areas in cancer research. Tumor cells are no longer viewed merely as genetically altered entities, but rather as highly plastic systems in which metabolic rewiring and epigenetic remodeling cooperate to sustain proliferation, adaptation, survival, and therapeutic resistance. This bidirectional relationship has progressively revealed how metabolites can directly shape chromatin architecture and gene expression, while epigenetic programs, in turn, orchestrate metabolic phenotypes that support tumor progression.

The Research Topic Epigenetics and Metabolism: A Closed Liaison in Tumor Cells was conceived with the aim of gathering multidisciplinary contributions focused on this intricate and fascinating crosstalk. The articles collected in this issue highlight how metabolic intermediates, chromatin modifications, non-coding RNAs, and transcriptional regulators converge to modulate cancer cell fate and tumor evolution. Together, these works provide a contemporary overview of the epi-metabolic networks underlying malignancy and identify emerging therapeutic vulnerabilities.

Among the contributions included in this Special Issue, the review by Bansal et al. discusses the epigenetic consequences of reducing sugars and glycation products in cancer, emphasizing how metabolic alterations associated with glycation may profoundly influence chromatin organization and transcriptional regulation. Their work reinforces the concept that metabolic byproducts are not passive consequences of tumor metabolism but active signaling molecules capable of driving oncogenic programs.

The Perspective article by Atlante et al. further expands this concept by focusing on the relationship between metabolic rewiring and chromatin methylation. Their work highlights how metabolites derived from altered tumor metabolism can directly influence methylation-dependent chromatin states, ultimately affecting tumor plasticity and adaptation. Importantly, this contribution underlines the therapeutic potential of targeting epi-metabolic dependencies in cancer cells.

Another important aspect addressed in this Research Topic concerns the role of non-coding RNAs in coordinating metabolic and epigenetic responses in tumors. The Mini Review by Mangraviti and Castelli summarizes emerging evidence showing that microRNAs, lncRNAs, and other classes of ncRNAs act as central mediators linking metabolic stress and epigenetic regulation in cancer cells. This layer of regulation adds further complexity to tumor adaptability and suggests novel opportunities for biomarker discovery and therapeutic intervention.

Finally, the review by Akdaş et al. examines the role of the NAD(H)-dependent corepressor CTBP1 as a molecular sensor integrating metabolic cues with transcriptional and oncogenic programs. This work exemplifies how metabolic cofactors can directly regulate epigenetic and transcriptional machinery, reinforcing the concept that metabolism and epigenetics cannot be studied as isolated processes in cancer biology.

Collectively, the studies presented in this Research Topic support the idea that the metabolic state of tumor cells is deeply interconnected with epigenetic plasticity. Metabolites such as acetyl-CoA, S-adenosylmethionine, α-ketoglutarate, succinate, fumarate, and NAD+ are now recognized as crucial regulators of chromatin-modifying enzymes and transcriptional activity. Conversely, epigenetic alterations reshape glucose, lipid, and amino acid metabolism, thereby sustaining the malignant phenotype and contributing to intratumoral heterogeneity and therapy resistance (Dai et al., 2024).

Despite significant progress, many questions remain unresolved. Future studies will need to better define the temporal dynamics of epi-metabolic interactions, their tumor-specific context dependency, and their contribution to metastatic dissemination and drug resistance. In parallel, advances in single-cell and spatial multi-omics technologies will likely provide unprecedented opportunities to dissect the heterogeneity of these processes within the tumor microenvironment. We believe that deciphering this “closed liaison” will not only improve our understanding of tumor biology but also pave the way toward innovative therapeutic strategies capable of simultaneously targeting metabolic and epigenetic vulnerabilities in cancer cells.
